# Optimization of De Novo Short Read Assembly of Seabuckthorn (*Hippophae* *rhamnoides* L.) Transcriptome

**DOI:** 10.1371/journal.pone.0072516

**Published:** 2013-08-21

**Authors:** Rajesh Ghangal, Saurabh Chaudhary, Mukesh Jain, Ram Singh Purty, Prakash Chand Sharma

**Affiliations:** 1 University School of Biotechnology, Guru Gobind Singh Indraprastha University, Dwarka, New Delhi, India; 2 National Institute of Plant Genome Research, New Delhi, India; Harbin Institute of Technology, China

## Abstract

Seabuckthorn ( *Hippophae* *rhamnoides* L.) is known for its medicinal, nutritional and environmental importance since ancient times. However, very limited efforts have been made to characterize the genome and transcriptome of this wonder plant. Here, we report the use of next generation massive parallel sequencing technology (Illumina platform) and *de novo* assembly to gain a comprehensive view of the seabuckthorn transcriptome. We assembled 86,253,874 high quality short reads using six assembly tools. At our hand, assembly of non-redundant short reads following a two-step procedure was found to be the best considering various assembly quality parameters. Initially, ABySS tool was used following an additive k-mer approach. The assembled transcripts were subsequently subjected to TGICL suite. Finally, *de novo* short read assembly yielded 88,297 transcripts (> 100 bp), representing about 53 Mb of seabuckthorn transcriptome. The average length of transcripts was 610 bp, N50 length 1198 BP and 91% of the short reads uniquely mapped back to seabuckthorn transcriptome. A total of 41,340 (46.8%) transcripts showed significant similarity with sequences present in nr protein databases of NCBI (E-value < 1E-06). We also screened the assembled transcripts for the presence of transcription factors and simple sequence repeats. Our strategy involving the use of short read assembler (ABySS) followed by TGICL will be useful for the researchers working with a non-model organism’s transcriptome in terms of saving time and reducing complexity in data management. The seabuckthorn transcriptome data generated here provide a valuable resource for gene discovery and development of functional molecular markers.

## Introduction

Seabuckthorn ( *Hippophae* *rhamnoides* L.) is a hardy, deciduous shrub of family Elaeagnaceae. The plant is a wind pollinated dioecious with a diploid chromosome number of 24 [1]. Its natural habitat extends widely across Europe and Asia in countries like China, Mongolia, India, Russia, Sweden, Finland and Norway [2,3]. In India, seabuckthorn is generally found growing in hilly tracks of Ladakh in Jammu and Kashmir, Lahaul-Spiti in Himachal Pradesh and parts of Uttarakhand, Arunachal Pradesh and Sikkim [4]. All parts of the seabuckthorn plant are known for their medicinal value. Seabuckthorn has been used in traditional medicinal systems in Tibet, Mongolia, Uzbekistan, Pakistan, Turkey, China, Russia and India to treat bowel irregularities, gastric ulcers, skin infection/wounds, influenza infections, cough and cold [5–8]. Apart from medicinal importance, seabuckthorn is also popular for its ornamental and environmental importance. It has long been recognized as an important plant for stabilization of mobile sand dunes [9]. The plant is known to harbor *Frankia* , a nitrogen fixing actinomycetes, in its roots and therefore has been used in land reclamation [10]. Due to its high nutritional and medicinal value, seabuckthorn has attracted focus of agricultural scientists for domestication and breeding programmes. Surprisingly, limited information about seabuckthorn genome and transcriptome is available in public domain. This is reflected by repository of a small number of seabuckthorn ESTs (3412) in the dbEST of NCBI [11] and a solitary recent report of 454 sequencing based transcriptome profiling of seabuckthorn berries [12].

During past three decades, Expressed Sequence Tags (ESTs) have played a significant role in gene discovery and gene function analysis, particularly for non-model organisms. ESTs generated from Sanger’s sequencing approach have longer read lengths facilitating easy assembly into longer consensus sequences for further downstream analysis. However, over the last few years, new high-throughput and cost effective sequencing technologies have become available which greatly outperform the standard Sanger technology in terms of massive data generation at a much reduced cost and labor. Ultra high-throughput RNA sequencing has allowed transcriptome analysis in several species and offered an attractive approach for qualitative and quantitative analysis of the whole transcriptome. Next Generation Sequencing (NGS) technologies are now increasingly been considered to be an alternative to microarrays [13] for analyzing differential gene expression also. Among NGS technologies, Roche-454 platform has been widely used in transcriptome sequencing of various non-model organisms [14,15] due to its ability to generate relatively long reads which greatly facilitates *de novo* assembly as compared to Illumina platform and ABI SOLiD system. Though advancement with regard to increase of read length in Illumina platform reads is underway, several new *de novo* assembly tools are being developed to assemble short sequence reads generated by these NGS platforms. However, performance of these assembly tools across various data sets has been investigated in a relatively few studies only [16–18].

Prior knowledge of the efficiency of different *de novo* short read assembly tools is useful while undertaking a project on transcriptome analysis in an unexplored species. In the present study, we have optimized *de novo* transcriptome assembly of short reads generated from Illumina HiSeq 2000 platform using six frequently used short read assemblers and following two different strategies to select the best transcriptome assembly obtained.

## Materials and Methods

### RNA Isolation, Illumina Sequencing and Quality Control

Seabuckthorn leaf and root tissues were harvested from the seedlings grown in a plant growth chamber as mentioned in Ghang et al. al. 2012 [11]. RNA was isolated as described previously by Ghang et al. al. 2009 [19]. RNA samples having more than 8.0 RIN (RNA Integrity Number) value were used for further processing. mRNA was purified from 3.0 µg of total RNA using oligo(dT) beads and fragmented to generate short mRNAs. Taking these short fragments as template, first strand cDNA was synthesized using random hexamer primers. Purified double stranded cDNAs containing sequencing adaptors were sequenced using Illumina HiSeq 2000. Construction of leaf and root cDNA libraries and their paired end sequencing was out sourced to a commercial service provider, Ocimum Biosolution Pvt. Ltd., Hyderabad. Various quality control checks on the short reads were performed using NGS QC Tool Kit [20].

### Availability of Short Read Data

The Illumina short reads generated in this study have been submitted to NCBI’s Short Read Archive (SRA) with study accession number SRP011938, containing seabuckthorn leaf and root sample data under the accession numbers SRS304528 and SRS304529, respectively.

### De novo Short Read Assembly

In this study, we systematically studied and compared the performance of six commonly used *de novo* short read assembly tools including Velvet [21], Oases [22], ABySS [23], SOAPdenovo [24], CLC Genomics Workbench (commercially available) and Trinity [25] following different approaches. Most of these assemblers are based on de Bruijn graphs wherein sequence reads are broken into smaller sequences of DNA, referred to as k-mers, where k denotes the length of these smaller sequences [26].

**Figure 1:**
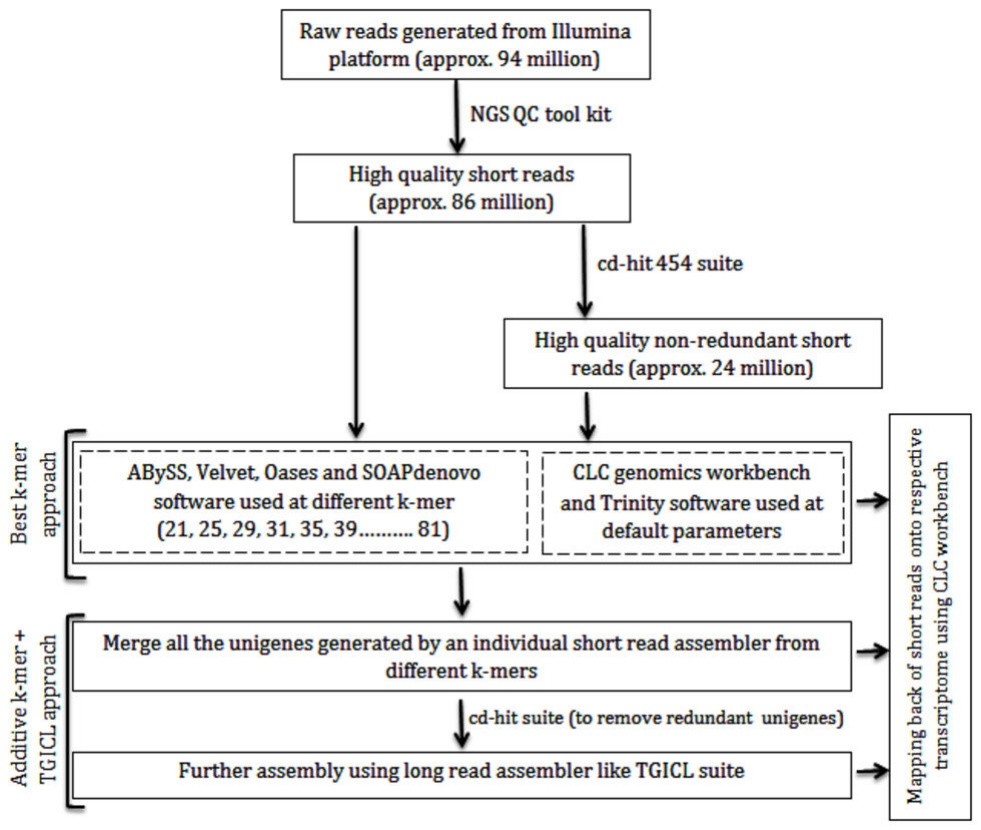
Flow chart showing the strategy used for generation of *de novo* short read transcriptome assembly.

*De novo* transcriptome assembly was performed following two different approaches. The outline of the methodology employed in the present study has been shown in the flow chart (Figure 1). In the first approach (best k-mer strategy), redundant (approx. 86 million) and non-redundant (approx. 24 million) high quality short reads were assembled using short read assemblers mentioned above. The k-value ranged from 21 to 81 for Velvet, Oases, ABySS and SOAPdenovo, whereas CLC and Trinity softwares were run at default settings. The best k-mer assembly was identified on the basis of various assembly parameters. In the second approach (additive k-mer followed by TGICL), a two-step strategy was employed. Initially, contigs generated for all k-mer values by respective assembler were merged and redundant sequences were removed by cd-hit tool [27]. The non-redundant contigs thus generated were assembled again using TGICL suite [28], as TGICL suite is effective in assembly of long reads rather than short sequences generated by NGS platforms. To check the integrity of assembled transcriptome generated using various assembly tools and following different strategies, high quality short reads were mapped back onto respective assembled *Unigenes* using CLC workbench software and compared for total mapped back reads and unique mapped back reads. Total mapped back reads depict the number of reads involved in the formation of *Unigenes* , while uniquely mapped back reads signifies the number of short reads, each showing single target in the assembled transcriptome. Ideally, assembly finalized for further annotation should have high percentage of total as well as uniquely mapped back reads. Various other parameters like total number of contigs (> 100 bp), N50 length and average contig length were also taken into consideration as a function of k-mer length to select the best possible transcriptome assembly. N50 length is a weighted median statistics such that 50% of the entire assembly is contained in contigs equal to or larger than this value. Since higher N50 length indicates better performance of the assembly tool, more weightage was given to N50 value rather than average read length wherever a marginal difference was present in average read length of compared assemblies.

### Similarity Search and Functional Annotation of Unigenes

Similarity search is one of the very first and easiest computational approaches for annotation of any transcriptome dataset. BLASTX module of the BLAST tool pack was employed for functional annotation because BLAST [29] is the most commonly used tool considering the statistical model applied for measuring the significance of local sequence similarities and computational speed. Only the best hit results were extracted and hits with an E-value <1E-06 were considered to be significant. Gene Ontology (GO) annotations for molecular function, biological process, and cellular component categories were based on the BLASTX hit from BLAST2GO suite [30].

### In silico Mining for Simple Sequence Repeats and Transcription Factors

Seabuckthorn transcriptome was screened for the presence of microsatellites or simple sequence repeats (SSRs) using MISA (MIcroSAtellite) [31], a perl script, using default parameters. The number of repeating units taken in the present study was atleast ten for mono-nucleotides, six for di-nucleotides, five for tri-nucleotides, five each for tetra-, penta- and hexa-nucleotides. For the identification of transcription factors represented in seabuckthorn transcriptome, transcripts were searched against all the transcription factor protein sequences available at Plant Transcription Factor Database [32].

## Results and Discussion

### Next Generation (Illumina) Sequencing and Quality Control

Recent advances in NGS technologies have markedly increased the amount of data generated and significantly reduced the sequencing cost. Although continuous strive is on to achieve long read lengths, it is still shorter when compared to that of Sanger sequencing technology. However, the field is undergoing revolutionary changes providing gradual improvement and advancements towards development of a perfect transcriptome assembly. In this study, a total of 94,013,936 (approx. 94 million) raw short reads (91 bp) were obtained from seabuckthorn leaf and root tissue libraries using Illumina HiSeq 2000 platform. The short reads were first filtered using NGS QC toolkit to remove low-quality reads and those having primer/adaptor contaminations. This exercise yielded a total of 86,253,874 high quality paired end reads. Although generation of single end reads is much cheaper than paired end reads in NGS techniques, paired end reads are important for *de novo* assembly due to their linked nature [33]. A summary of short read data (raw and filtered) generated for seabuckthorn transcriptome in this study has been presented in Table 1. As evident from this tabulated summary, collectively for both the tissues, more than 90% of the actual short reads were retained as high quality reads signifying the use of good quality RNA for next generation sequencing.

**Table 1:** Summary of Illumina HiSeq 2000 short read data used for seabuckthorn transcriptome assembly.

**Data item**	**Leaf**	**Root**
Total number of reads	44791536	49222400
Read length	91	91
Total number of HQ reads	41067522	45186352
Percentage of HQ reads	91.69%	91.80%
Total number of bases	4031238240 (4.0 Gb)	4430016000 (4.4 Gb)
Total number of bases in HQ reads	3696076980 (3.7 Gb)	4066771680 (4.0 Gb)
Total number of HQ bases in HQ reads	3619124516	3985259341
Percentage of HQ bases in HQ reads	97.92%	98%

HQ stands for high quality obtained after running NGS QC toolkit on raw data

For short read assemblers that perform assembly as a function of k-mer, lower the k-value, higher is the computer’s RAM requirement. Thus for RAM intensive assembly tools, high quality reads obtained from Illumina platform (approx. 86 million) were fed to cdhit-454 suite to remove redundancy resulting in the generation of 24,405,576 (approx. 24 million) non-redundant short reads. The assembly obtained from this non-redundant dataset has been mentioned as NR in the figures and following text.

### De novo Short Read Assembly

*De novo* assembly was performed following two strategies. In the first approach (best k-mer strategy), high quality reads (all approx. 86 million and non-redundant approx. 24 million) were assembled separately using Velvet, Oases, ABySS and SOAPdenovo programs at different k-mer lengths ranging from 21 to 81, whereas CLC Genomics Workbench and Trinity were used at default parameters (Table S1). A comparative analysis of assemblies generated by different assembly tools used in the present study revealed that best k-mer value varied for different tools. For example, in the case of Oases, decrease in k-mer value (at k=29-35) showed improved assembly statistics. On the other hand, in case of Velvet and SOAPde novo, increase in k-mer value (at k=69-79) led to the improvement in the generated assembly. Moreover, in case of ABySS the mid k-mer values were found to be the best as a gradual increase in assembly statistics up to k=55-59 (redundant sequences) and k=45-49 (non-redundant sequences) was observed that gradually decreased till end point k-mer (Table S1). However, on the basis of various parameters as a function of k-mer length as mentioned in material and methods, assembly generated by Oases at k=31 taking non-redundant short read dataset (NR-Oases) was considered to be the best among assemblies generated by first strategy (best k-mer strategy) with average read length of 794 bp and N50 read length of 1378 bp (Table 2; Table S1). Moreover, among all the short read assembler used in our study, Oases was found to be the most RAM intensive as about 512 GB RAM was required to perform assembly at k=21 using 86 million redundant high quality reads as an initial input.

**Table 2:** Comparison of different short read assemblers and strategies employed on the basis of various assembly parameters.

**Intial input reads**		**Redundant short reads (86 million)**						**Non-redundant short reads (24 million)**					
**Short Read Assembler**		**Velvet**	**Oases**	**ABySS**	**SOAP**	**CLC**	**Trinity**	**Velvet**	**Oases**	**ABySS**	**SOAP**	**CLC**	**Trinity**
**Assembly Parameters**	**Best k-mer**	**(K=71)**	**(K=31)**	**(K=55)**	**(K=71)**	**Default**	**Default**	**(K=71)**	**(K=31)**	**(K=49)**	**(K=71)**	**Default**	**Default**
**Total number of *Unigenes* **		77022	62804	102826	97306	279673	240723	72302	55238	139923	83813	283777	234329
**Maximum read length**		3293	8567	6650	3548	7070	14182	4182	8849	5001	3793	6257	9473
**Average read length**		378	733	698	330	310	548	403	794	335	368	300	460
**N50 read length**		454	1314	1144	429	486	1284	485	1378	482	474	434	791
**Total map back reads (%)**		73.79	68.28	89.21	76.65	85.77	90.8	77.69	72.99	69.42	81.55	83.41	90.24
**Unique map back reads (%)**		71.15	66.95	41.93	73.2	84.74	52.27	75.5	72.16	68.91	79.01	82.64	55.95
**Short Read Assembler**	**Additive k-mer**	**Velvet**	**Oases**	**ABySS**	**SOAP**	**CLC**	**Trinity**	**Velvet**	**Oases**	**ABySS**	**SOAP**	**CLC**	**Trinity**
**Total number of *Unigenes* **		347958	115006	111040	314576	279673	240723	332830	97691	167892	295569	283777	234329
**Maximum read length**		4668	10607	7659	4654	7070	14182	4931	10387	6552	5384	6257	9473
**Average read length**		321	798	804	339	310	548	330	856	421	352	300	460
**N50 read length**		419	1504	1317	458	486	1284	435	1533	647	484	434	791
**Total map back reads (%)**		93.73	94.89	94.14	94.07	85.77	90.8	94.53	94.77	94.05	95.05	83.41	90.24
**Unique map back reads (%)**		66.1	46.97	45.27	69.28	84.74	52.27	66.17	48.9	71.54	70.28	82.64	55.95
**Short Read Assembler**	**Additive + TGICL**	**Velvet**	**Oases**	**ABySS**	**SOAP**	**CLC**	**Trinity**	**Velvet**	**Oases**	**ABySS**	**SOAP**	**CLC**	**Trinity**
**Total number of *Unigenes* **		228750	101055	85902	211863	270245	212093	219896	86124	88297	202713	274248	202089
**Maximum read length**		7608	16199	14243	11102	9026	14182	9030	17565	10252	9015	6257	9474
**Average read length**		374	871	930	396	317	475	385	932	610	407	307	444
**N50 read length**		631	1606	1539	690	513	1035	658	1629	1198	725	456	766
**Total map back reads (%)**		93.43	94.79	94.08	93.69	85.83	91.66	94.22	94.76	93.21	94.77	83.46	91.03
**Unique map back reads (%)**		90.07	50.25	54.11	90.67	85.15	67.2	90.7	52.15	91.03	91.16	83.07	70.15

In the second approach (additive k-mer followed by long read assembler TGICL), a two-step strategy was employed. In the first step, contigs obtained from all the k-mer of respective assembler were merged and redundant sequences removed. In the second step, non-redundant contigs thus obtained in the above step were then again assembled using long read assembler TGICL suite. Groba and Burgos [34] suggested additive multiple-k method to be a better approach than single best k-mer as it improves transcript diversity and increases contiguity. We also observed that additive k-mer approach increased the average and N50 read length to a large extent thereby improving the transcriptome assembly significantly (Table 2). Moreover, except for CLC, assemblies obtained from all other short read assemblers showed more than 90% of the short reads mapping back onto their respective transcriptome assembly (Table 2). In contrast, employing best k-mer approach, only Trinity assembly had more than 90% mapped back reads (Table 2). This observation further supports the use of additive k-mer approach over best k-mer to get a better assembly. However, when percentage of uniquely mapped reads was compared, it showed a completely different picture. Although the average read length, N50 length and percentage of total mapped back reads increased, there was a sharp decrease in the percentage of unique mapped back reads (Table 2) thereby suggesting redundant use of short reads in final assembly and formation of chimeric *Unigenes* as one short read must map back to a single locus and take part in the formation of a single transcript. To overcome this problem and further improve assembly statistics, transcriptome assembly obtained from additive k-mer approach was subjected to long read TGICL assembler. This step further increased the average read length and N50 length of all the assemblies. Although there was a marginal increase in the percentage of total mapped reads, a marked increase in the percentage of unique mapped back reads was observed in some of the assemblies (Table 2). A preliminary examination suggested the assembly generated by Oases taking non-redundant short read dataset to be the best (NR-Oases) as it had highest average read length, N50 length and total mapped back reads (Table 2). Nevertheless, when uniquely mapped reads were taken into consideration, only 52% (44,978,322) of the total reads uniquely mapped back onto NR-Oases assembly (Table 2), thus indicating the possibility of generation of chimeric *Unigenes* . Therefore, this assembly was not used for further analysis. On the basis of mapping results, NR-SOAPdenovo and NR-ABySS assemblies were found to be most impressive as more than 90% of the short reads uniquely mapped back onto the transcriptome (Table 2). However, when other parameters like total number of contigs (88,297), N50 read length (1198 bp) and average contig length (610 bp) were considered, assembly generated by NR-ABySS was found to be the best (Table 2; File S1). Although different strategies employed in this study gradually increased the N50 and average read length, use of only additive k-mer approach decreased the proportion of unique mapped back reads with respect to total mapped back reads. However, the use of TGICL on additive k-mer assemblies increased the map back proportion to a large extent suggesting the use of long read assembly tools on data initially generated by preliminary short read assemblers.

### Similarity Search and Functional Annotation of Unigenes

Keeping a significant E-value cutoff of < 1E-06, we used BLASTX algorithm to annotate 88,297 seabuckthorn transcripts (obtained from NR-ABySS following second strategy) against non-redundant (nr) protein database of NCBI. A total of 41,340 (46.8%) seabuckthorn transcripts were found to have a significant hit with protein entries present in the NCBI database (Table S2). This figure is comparable with that reported by Fatima et al. 2012 [12] where 46% of seabuckthorn transcripts obtained from 454 sequencing showed similarity to existing proteome database. However, in our previous pilot study based on a small seabuckthorn dataset comprising of 1,665 *Unigenes* , we were able to annotate nearly 77% of the *Unigenes* [11].

**Figure 2:**
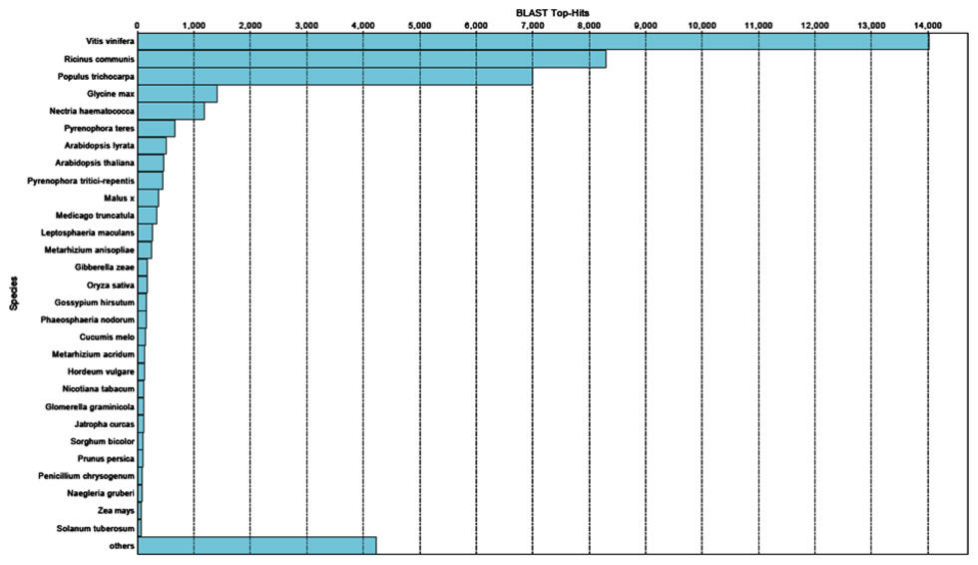
Top hit species representation of seabuckthorn transcriptome based on sequence similarity search (BLASTX).

In accordance with our previous study [11] and findings of Fatima et al. 2012 [12], we also observed significant sequence homology between *Vitis vinifera* (grapes) and seabuckthorn. According to amino acid homology, species showing top-hits at NCBI database included *V. vinifera, Ricinus communis,* *Populus* *trichocarpa* and *Glycine max* (Figure 2), in that order. We used BLAST2GO tool to assign gene ontology (GO) terms associated with top 20 BLAST hits for each transcript. Of the 41,340 sequences having BLAST hit (E-value cutoff of < 1E-06), 38,830 had at least one GO term associated with each of them representing 43.9% of the seabuckthorn transcriptome. Seabuckthorn transcripts could be grouped in different categories viz. biological process (Figure S1), cellular component (Figure S2) and molecular function (Figure S3) depicting a broad spectrum of the analyzed transcriptome. In the category of biological process, the largest groups were “primary metabolic process”, “cellular metabolic process” and “biosynthetic process”. However, presence of groups like “cellular response to stimulus”, “response to external stimulus”, “response to biotic stimulus”, response to endogenous stimulus”, “response to abiotic stimulus” and “response to stress” in our dataset indicated that a large number of transcripts are expressed in response to environmental stresses (Figure S1). In the category of molecular function, *Unigenes* with “catalytic activity”, “protein binding” and “nucleotide binding” formed the largest groups (Figure S3).

### In silico SSR Screening and Detection of Transcription Factors

EST based markers, owing to high polymorphism, abundance and ease in development are important resource for determining functional genetic variation. Mining of SSRs from seabuckthorn transcriptome was performed using MISA search tool at default parameters. A total of 13,299 SSRs were identified in 10,980 (12.4%) seabuckthorn transcripts (Table 3). Though mononucleotide SSRs represented the largest fraction (56.4%) but due to the presence of polyA tail in eukaryotic mRNA which confounds with microsatellite repeat, these *in silico* mined mono-nucleotide SSRs should be considered with care for further analysis. Among the rest, dinucleotide repeats (21.5%) were found to be most common EST-SSR in seabuckthorn transcriptome followed by trinucleotide repeats (18.9%) which is in accordance with our previous report [11] on a limited number of 1,665 *Unigenes* . Among dinucleotide repeats, AG/CT accounted for the highest occurrence (67%) in seabuckthorn transcriptome followed by AT/TA (23%), AC/GT (9.5%) and marginal CG/CG (0.2%). While in case of trinucleotide repeats, occurrence of various constituting repeats was uniform except for AAG/CTT showing higher frequency (38%) and CCG/CGG showing least abundance (0.4%).

**Table 3:** Data on distribution of Simple Sequence Repeats (SSRs) in seabuckthorn transcriptome.

**Data item**	**Number (%)**
Total number of sequences screened	88297
Total number of identified SSRs	13299
Number of sequences containing SSRs	10980 (12.4)
Number of sequences containing more than one SSR	1850 (16.8)
Number of SSRs present in compound formation	1099 (8.2)
**Relative distribution of different repeat types**	
Mono-nucleotides	7502 (56.4)
Di-nucleotides	2860 (21.5)
Tri-nucleotides	2520 (18.9)
Tetra-nucleotides	213 (1.6)
Penta-nucleotides	62 (0.46)
Hexa-nucleotides	142 (1.06)

**Figure 3:**
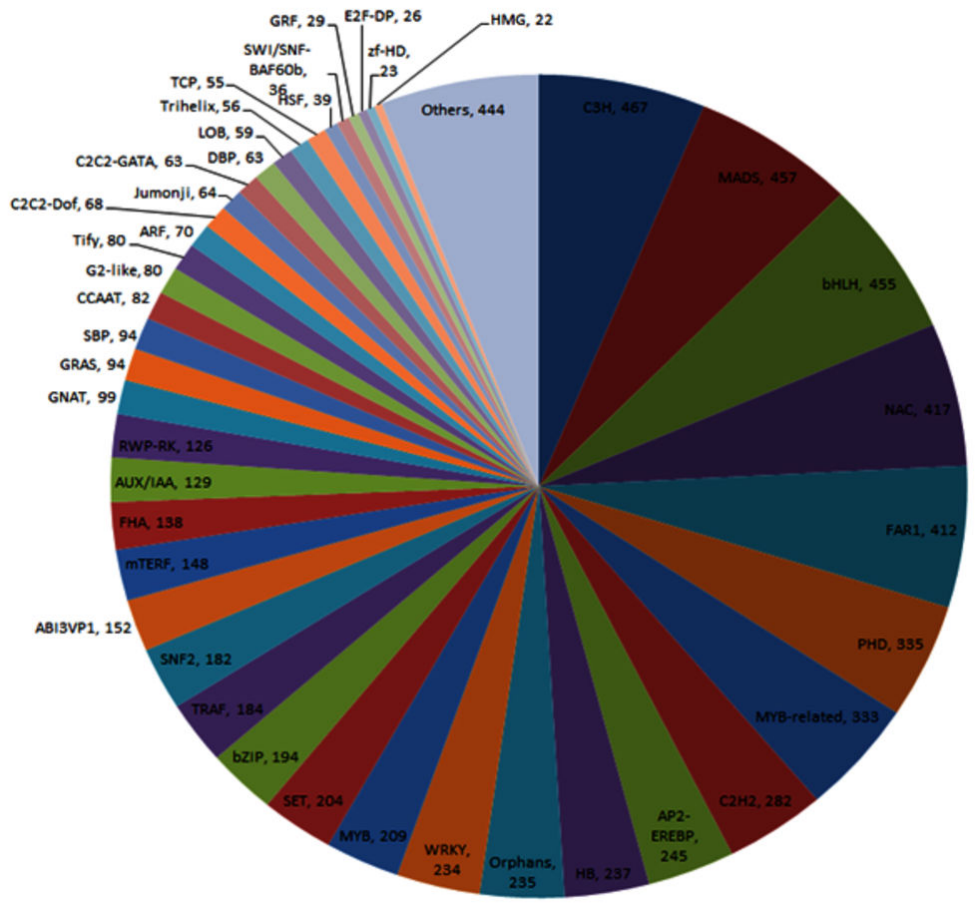
Distribution of seabuckthorn transcripts in different transcription factor families.

The seabuckthorn transcriptome generated in this study was also screened for the presence of transcription factors by sequence comparison to known transcription factor gene families. In total, 7,421 putative seabuckthorn transcription factor genes, distributed in at least 80 different families, were identified representing 8.4% of seabuckthorn transcripts. The most frequent transcription factors belonged to C3H, MADS, bHLH, NAC, and FAR1 families (Figure 3). Transcription factors involved in abiotic stress tolerance have also been identified in our dataset. Earlier we reported expression analysis of HMG transcription factor in response to freeze tolerance in seabuckthorn [11].

## Conclusions

Next generation sequencing technologies are becoming increasingly affordable, accessible and robust for non-model organisms also enabling faster and cheaper data generation. However, task of extracting meaningful information from such a huge amount of data has become more challenging. Here, with the objective to generate transcriptome of a non-model organism, seabuckthorn, using Illumina short reads, we compared the utility of six *de novo* short read assembly tools following different approaches. The study suggested removal of redundancy in short read dataset prior to assembly by short read assembler ABySS following additive k-mer approach. Subsequent assembly by long read assembler TGICL yielded the optimum assembly. Looking at the prospects of using TGICL after initial short read assemblers, as observed in the present study, it is recommended to use long read assemblers after *de novo* short read assemblers in future transcriptome assembly projects. Functional annotation of seabuckthorn transcriptome revealed conservation of genes involved in various biological processes. Our study demonstrates that NGS technique is a powerful tool for gene function discovery even in organisms with no annotated genomes.

## Supporting Information

Figure S1 **Gene Ontology classification of seabuckthorn *Unigenes* on the basis of their role in biological processes.**
(TIF)

Figure S2 **Gene Ontology classification of seabuckthorn *Unigenes* on the basis of their occurrence in different cellular components.**
(TIF)

Figure S3 **Gene Ontology classification of seabuckthorn *Unigenes* on the basis of their molecular function.**
(TIF)

File S1 **Unigene sequences of *de novo* assembled seabuckthorn transcriptome.**
(7Z) Click here for additional data file.

Table S1 **Assembly statistics of various short read assemblers at different k-mers.**
(XLS)

Table S2 **Functionally annotated seabuckthorn *Unigenes* on the basis of BLASTX results.**
(XLSX)
