# Correction: Optimization of De Novo Short Read Assembly of Seabuckthorn (*Hippophae* *rhamnoides* L.) Transcriptome

**DOI:** 10.1371/annotation/8af6a452-11e9-45c1-995d-7fee9b0456eb

**Published:** 2013-08-29

**Authors:** Rajesh Ghangal, Saurabh Chaudhary, Mukesh Jain, Ram Singh Purty, Prakash Chand Sharma

There was an error in the citation. The correct citation is available below.

Ghangal R, Chaudhary S, Jain M, Purty RS, Sharma PC (2013) Optimization of De Novo Short Read Assembly of Seabuckthorn (*Hippophae rhamnoides* L.) Transcriptome. PLoS ONE 8(8): e72516. doi:10.1371/journal.pone.0072516 

