# Supplementary figures and images for: Optimization of De Novo Short Read Assembly of Seabuckthorn (Hippophae rhamnoides L.) Transcriptome
Source: PLoS One. 2013 Aug 21;8(8):e72516. doi: 10.1371/journal.pone.0072516 (PMC3749127; doi:10.1371/journal.pone.0072516)

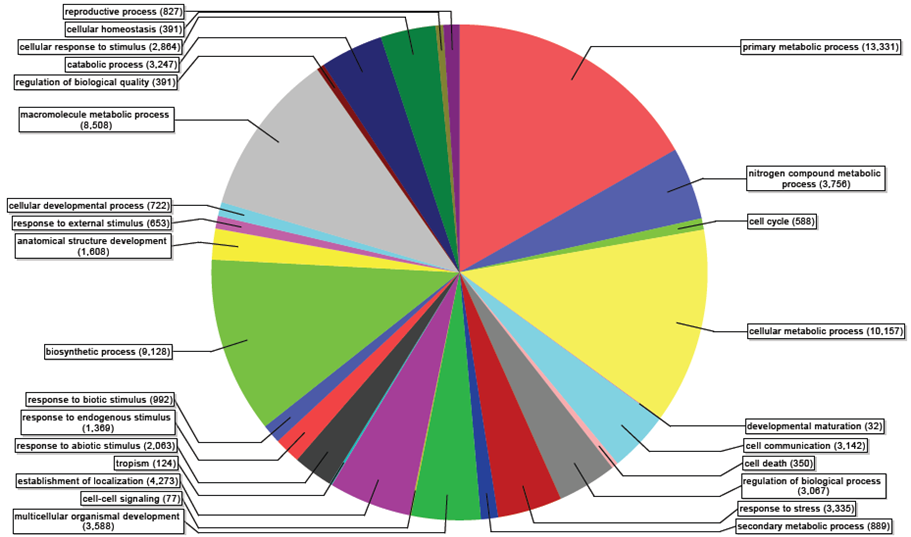

Supplement: Figure S1 — Gene Ontology classification of seabuckthorn Unigenes on the basis of their role in biological processes. (TIF) [file pone.0072516.s001.tif]

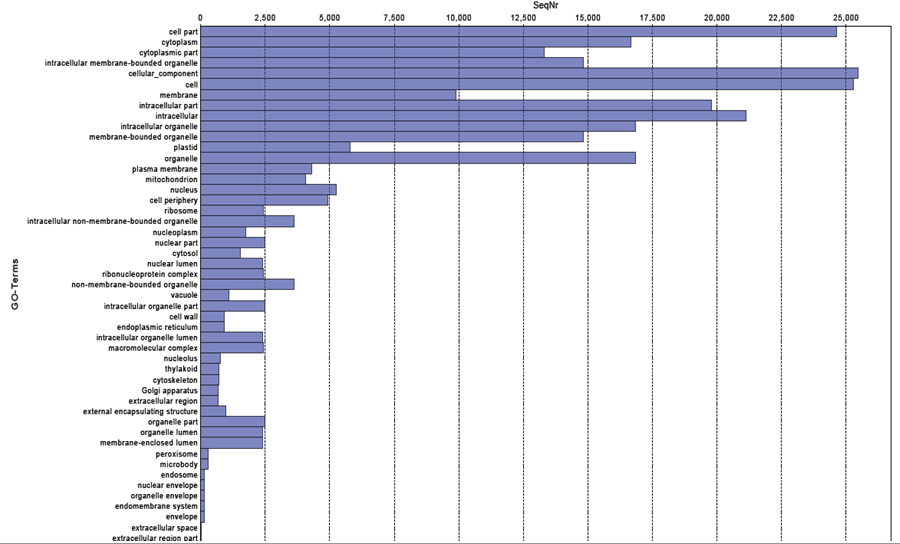

Supplement: Figure S2 — Gene Ontology classification of seabuckthorn Unigenes on the basis of their occurrence in different cellular components. (TIF) [file pone.0072516.s002.tif]

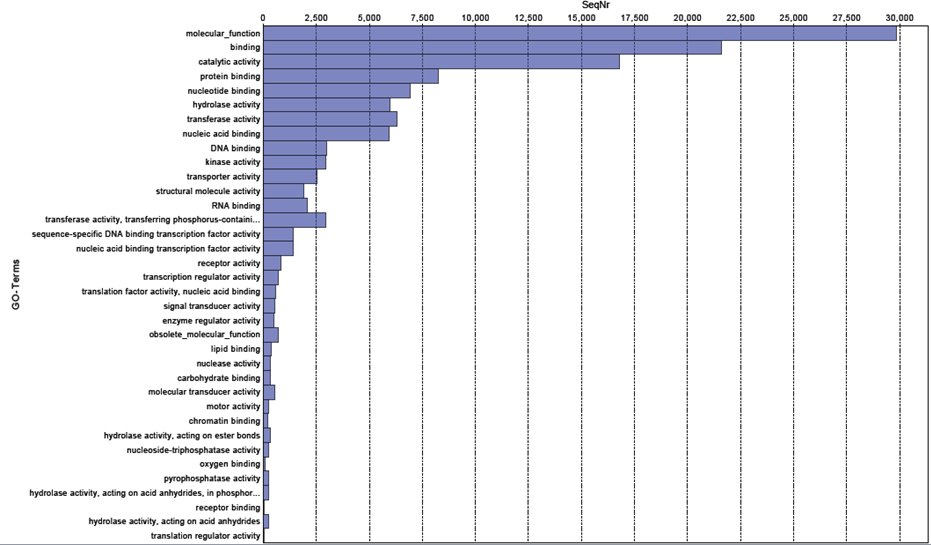

Supplement: Figure S3 — Gene Ontology classification of seabuckthorn Unigenes on the basis of their molecular function. (TIF) [file pone.0072516.s003.tif]
